# Effect of altitude of coffee plants on the composition of fatty acids of green coffee beans

**DOI:** 10.1186/s13065-020-00688-0

**Published:** 2020-05-12

**Authors:** Girmay Tsegay, Mesfin Redi-Abshiro, Bhagwan Singh Chandravanshi, Estifanos Ele, Ahmed M. Mohammed, Hassen Mamo

**Affiliations:** 1 Department of Chemistry, College of Natural Sciences, Addis Ababa University, P.O. Box 1176, Addis Ababa, Ethiopia; 2 Agricultural Quality Research Laboratory, Ethiopian Institution of Agricultural Research, P.O. Box 2003, Addis Ababa, Ethiopia; 3 Department of Microbial, Cellular and Molecular Biology, College of Natural Sciences, Addis Ababa University, P.O. Box 1176, Addis Ababa, Ethiopia

**Keywords:** *Coffea arabica*, Green coffee beans, Fatty acids, Effect of altitudes, Ethiopia

## Abstract

**Background:**

The fatty acids of green coffee beans are one of the major components that determine the quality of coffee. Fatty acids composition of green coffee beans is affected by soil composition and altitude of coffee plants. This study was aimed to evaluate the effect of altitude of the coffee plants on the composition of fatty acids in green coffee beans.

**Methods:**

Fatty acids contents of 40 green coffee beans samples collected from the coffee plants grown at different altitudes (group 1: 1500–1700, group 2: 1701–1900 and group 3: > 1900 m.a.s.l.) in Ethiopia were determined using gas chromatography-mass spectrometry (GC–MS). Chemometric data analyses were performed to determine the effects of altitude on the fatty acid composition of the green coffee beans.

**Results:**

The green coffee beans contained main saturated fatty acid, palmitic acid with an average value of 55.5 mg/g and unsaturated fatty acid, linoleic acid with an average value of 51.6 mg/g. The other major constituents of fatty acids present in green coffee beans were stearic and oleic acids with the value of 12.3 mg/g and 8.92 mg/g, respectively. Palmitic acid content in lowland green coffee beans is significantly different than in the other two regions. On the other hand, stearic and oleic acids contents in the green coffee beans did not show a significant difference between the three topographical regions. While linoleic acid content in the green coffee beans showed significant difference between group 1 and 3 but did not show significant differences between group 1 and 2 and between group 2 and 3. The four major fatty acids, palmitic (R = − 0.574), linoleic (R = − 0.506), stearic (R = − 0.43) and oleic acids (R = − 0.291) in green coffee beans showed a moderate negative correlation with the altitude of coffee plants.

**Conclusion:**

The fatty acids contents decreases with increasing altitude of the coffee plants and hence affects the quality of coffee. The fatty acid composition of green coffee beans can also be used to determine the topographical origin of coffee plants.

## Background

Coffee is the most valuable commodities in the world. It is cultivated in more than 80 countries in the tropical and subtropical regions of the world. Coffee is well adapted to different eco-physiological conditions of the tropics and subtropics [1–3]. The coffee (*Coffea arabica* L.) is the most important gift of Ethiopia to the world, which has wonderful economic and social impact on peoples of different geographical locations, cultural background, and psychological make-up. Coffee is appreciated international beverages; most of the peoples drink coffee every day, sometimes twice, or more times a day [4, 5]. It is a popularly consumed beverage with its extremely complex flavor. Coffee is the second largest traded commodity after oil in the world [6]. The genus *Coffea* is believed to comprises about 103 species worldwide, from these about 70 major species of the genus *Coffea* is found in the tropical area, but only three of them (*Coffea arabica*, *Coffea robusta*, and *Coffea liberica*) are cultivated for commercial coffee consumption [1, 7].

Arabica coffee produces superior quality than Robusta coffee [8, 9]. It dominates the world trade due to its superior quality and due to its pleasant taste, aroma, and stimulant effect [10]. Arabica coffee is highly watched due to its more superior organoleptic properties and it covers 64% of the world’s total coffee production. Robusta coffee has a slightly weaker taste and bitter compared to the Arabica coffee, and it covers 35% of the world production. Robusta coffee is used as a more economical substitution for Arabica coffee, but *Liberica* coffee is much less in demand and it covers much lesser amounts of the universal production. In general, the two species (Arabica and Robusta) are economically important [1]. They are the most important supplies in the international trade, for which highest quality is demanded [11].

Coffee quality is defined by its sensorial aspects, which are developed by the chemical prototypes found in fresh grains [12]. Quality and content of the chemical composition of coffee beans widely vary based on the species of coffee, altitude, soil, daily temperature variations, and the place of growing [13–17]. Coffee taste is different due to the presence of different volatile and nonvolatile chemical constituents.

Fatty acids are found in crude oil in the form of glycerides, sometimes present as free fatty acids. Fatty acids are a member of the larger class of ubiquitous lipids. Lipids are the most important components of coffee beans [18]. The coffee oil (lipids) mainly consists of triglycerides, sterols, tocopherols and the coffee characteristic diterpenes and their esters with fatty acids [19]. Many fatty acids are present in the oil extract from Arabica green coffee beans [9, 20]. The main fatty acids present in green coffee beans are linoleic and palmitic acids. The minor fatty acid compositions are myristic, palmitoleic, eicosenoic, behenic, linolenic and arachidic acids [20]. The fatty acids compositions are classified as saturated fatty acids, monounsaturated fatty acids and polyunsaturated fatty acids [21]. In general, the extracted coffee oil contained saturated fatty acids in the range of 49.4–59.2%, polyunsaturated fatty acids have the range 29.5–39.2% and monounsaturated fatty acids are in the range of 4.30–9.69 [22].

Coffee oil with the presence of linoleic acid is an excellent emollient, skin treatments and essential in human nutrition because of its uses to prostaglandin synthesis and other biological processes related to cell regeneration; its absence has been associated to dermatological disorders [22]. High concentrations of palmitic acid in the coffee oil provide good skin protection [18]. Green coffee beans contain high amounts of linoleic and palmitic acids.

Fatty acids are an important factor to assess the quality of the coffee. It is affected by altitude, soil, and the place where coffee plants are grown. Accordingly, comparison of fatty acids are used as a tool for classification purposes and fatty acids contents are used as a chemical descriptor to differentiate between coffee varieties and origins of the coffee [20, 23]. Recently some studies have been reported on the effect of altitude of the coffee plants on the chemical composition of green coffee beans. Bertrand et al. [13] have reported the comparison of bean biochemical composition and beverage quality of Arabica hybrids involving Sudanese-Ethiopian origins with traditional varieties at various elevations in Central America. Sherge et al. [24] have studied the influence of growing altitude, shade and harvest period on quality and biochemical composition of Ethiopian specialty coffee. Hagos et al. [3] have studied the correlation between caffeine contents in green coffee beans and altitudes of the coffee plants grown in southwest Ethiopia. Mintesnot and Dechassa [25] have studied the effect of altitude, shade, and processing methods on the quality and biochemical composition (caffeine, trigonelline, and chlorogenic acids) of green coffee beans in Ethiopia. Adem et al. [26] have reported the effect of altitude on biochemical composition (caffeine, chlorogenic acids and sucrose contents) and quality of green arabica coffee beans. Bodner et al. [27] have reported the effect of harvesting altitude on the aroma released by coffee powder. Gebrekidan et al. [28] have reported the influence of altitudes of coffee plants on the alkaloids contents of Ethiopian green coffee beans. However, there is no study on the specific impact of altitude on the fatty acid content of Ethiopian green coffee beans. Therefore, the aim of this study was to evaluate the effect of altitude of the coffee plants on the composition of fatty acids in the green coffee beans.

## Materials and methods

### Apparatus and instruments

An electronic grinder (Moulinex, SEB Group, Selongey, France), a 600 μm sieve (Chicago, ILL. 60656, USA), electronic balance (SP 1500, USA), Soxhlet extractor apparatus, rota evaporator (IKA, RV 10, USA), ^1^H and ^13^C NMR spectrometer (Bruker avance 400 MHz, USA), Agilent gas chromatograph equipped with a mass spectrometer detector and Agilent automatic injector spectrometer (Agilent Technologies, 7890A GC–MS, USA) were used.

### Chemicals

Dichloromethane ($\ge$ 97%, Sigma-Aldrich, USA) GC/MS grade, anhydrous methanol ($\ge$ 99.8%, Sigma-Aldrich, USA), ethanol ($\ge$ 98%, Sigma-Aldrich, USA), n-hexane ($\ge$ 97%, Sigma-Aldrich, USA), petroleum ether, concentrated sulfuric acid ($\ge$ 98%), anhydrous sodium sulfate (Fluka, Buchs, Switzerland), potassium hydroxide (Panreac, Barcelona, Spain), sodium carbonate, sodium hydrogen carbonate, sodium chloride (Manchester, UK), standards of decanoic acid ($\ge$ 99%, Sigma-Aldrich, USA) and oleic acid ($\ge$ 99%, Sigma-Aldrich, USA) were used as received.

### Sample collection

Green coffee beans samples were collected from different geographical origins of Ethiopia. Ethiopian coffee grows mainly in the administrative regions of Oromia, Harar and South Nations Nationalities and Peoples Region (SNNPR) [29]. For this study, 40 samples were collected from five different districts of these regions: namely: Gedeo/Yirgacheffe (15), Jimma/Gomma (12), Sidama (7), Bedele (2), Illubabur and Welega (4). Samples were collected from the coffee plants grown at three altitude ranges. Altitudes under study were: group 1: 1500–1700, group 2: 1701–1900 and group 3: ˃1900 m.a.s.l. The average annual temperature and rainfall were 21.5 °C and 1465 mm at group 1; 18.5 °C and 1525 mm at group 2; and 17.5 °C and 1700 mm at group 3, respectively. The samples were collected from the model farmers who produce a special cultivated coffee. The sample collection was done strategically with the District agricultural expert recommendation of the Ethiopian Institution of Agricultural Research and the model farmers were assigned by them. All the green coffee beans samples were obtained from ripe coffee cherries; they were processed by sun drying. 1 kg of green coffee beans were collected from different farmers from each sampling site and stored in paper bags under room temperature conditions. It was ground by an electronic grinder and sieved through the mesh size of 0.6 mm for the analysis.

### Extraction of lipids from green coffee beans

Total lipids were extracted from 20.0 g of powdered green coffee beans with hexane by refluxing in a Soxhlet apparatus according to Martín et al. [20] procedure with some modifications. A 20.0 g of powdered green coffee beans sample was weighed in timbel by electronic analytical balance; this was placed in chamber apparatus that was fitted with 250 mL round bottom flask containing 150 mL hexane and connected to the condenser of the Soxhlet extractor. The Soxhlet extractor was heated at 60 °C on a heating mantle for 6 h and cooled at room temperature. The extracted oil was dried by anhydrous sodium sulfate and filtered through Whatman filter paper (12 mm, dm). The solvent was evaporated by using a vacuum rotary evaporator. The crude oils were recovered after solvent evaporation at low temperature to remove residuals from solvents. Finally, the extracted oil was kept in a vial and stored in the refrigerator until the analysis.

### Derivatization of fatty acids

Fatty acid methyl esters were prepared by the transesterification method [30, 31] with some modification. Transesterification of oil sample was carried out in methanol using potassium hydroxide as a base. Fatty acid methyl ester were prepared by taking 1 g of coffee oil and dissolved with 6 mL of a methanolic solution of potassium hydroxide in 100 mL volumetric flask. The solution was heated at 60 °C boiling water bath for 60 min. The reaction mixture was removed from the water bath and allowed to cool to room temperature. 1 mL saturated sodium chloride solution was added to the cooled esterified solution and swirled gently several times. The methyl esters of fatty acids (FAMEs) produced was mixed with 30 mL of hexane and then the mixture was transferred to a separatory funnel and shaken for few minutes. The mixture was allowed to settle until it forms a layer, the distinct upper layer of methyl ester in n-hexane was separated carefully into 100 mL Erlenmeyer flask. To remove trace amount of water, anhydrous Na_2_SO_4_ was added to the hexane extract. The solution was filtered by Whatman filter paper and concentrated with rotary evaporator. The accuracy of the derivatization and analytical procedure was evaluated by validation with the standard of oleic acid. The percentage yield of esterified oleic acid methyl ester in reaction one was calculated using Eq. (1):

1 $${\text{Percentage yield}} = \frac{\text{Actual yield}}{\text{Theoretical yield}} \times 100$$

**Scheme 1:**
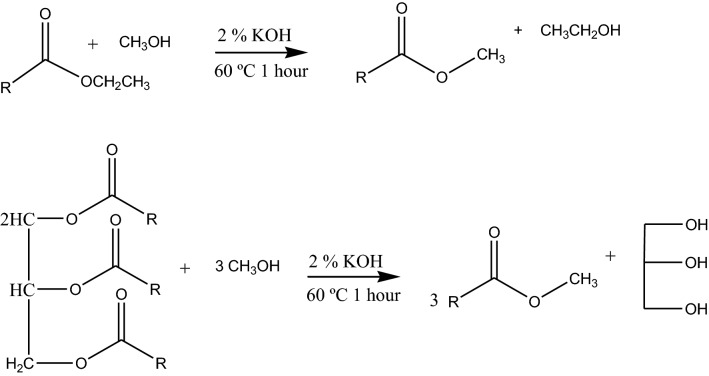
Transesterification of standard ethyl oleate to methyl oleate and that of the sample

The actual yield was obtained by direct weighing of the reaction product, but theoretical yield was calculated from the reaction equation (Scheme 1). Finally, the purity of the standard was characterized by ^1^H and ^13^C NMR spectroscopic data. This method was evaluated by the formation of methyl oleate from ethyl oleate according to the fatty acid methyl ester (FAME) formation procedure and the purity was characterized by ^1^H and ^13^C NMR spectroscopy.

### Determination of fatty acid composition

Fatty acid methyl esters obtained from the green coffee beans extracts were analyzed by GC–MS. It was prepared in the concentration of 9 µg/mL of the sample with 5 µg/mL of standard decanoic acid methyl ester. Samples were analyzed using GC–MS Agilent Technology 7820A GC and 5977E MSD systems equipped with auto sampler. Chromatographic separations were carried out using DB-1701 column with 30 m length, 0.25 mm internal diameter and 0.25 μm column phase thickness. Injection mode was split-less, helium was a carrier gas and 1 μL volume of the sample was injected to the inlet heated to 275 °C. An oven temperature condition was programmed as 60 °C for initial and hold for 2 min and reached up to 280 °C. The program was separated into the rate of 20 °C/min until it 200 °C and the rate of 3 °C/min up to it reaches 240 °C with zero hold time. Conditions used for the mass spectrometer were a source temperature of 230 °C, scan range 40–650 m/z, and operated in positive electron impact mode with ionization energy of 70 eV. Chromatographic and mass spectral data were processed by using the instrument built in software (MS ChemStation; Agilent Technologies, USA). For identification purposes, chromatograph library was used and its quantification was calculated by the internal standard with the relationship of relative response factors.

### Calculation of relative response factor (RRF)

To obtain the relative response factor (RRF) a known concentration of decanoic acid methyl ester and oleic acid methyl ester standards with 1:1 ratio were used. From these RRF was calculated using Eq. 2 [32, 33]:

2 $${\text{RRF }} = \frac{{{\text{P}}_{\text{ARS}} \times {\text{C}}_{\text{IS}} }}{{{\text{P}}_{\text{AIS}} \times {\text{C}}_{\text{RS}} }}$$

where P_ARS:_ the peak area of the reference standard by GC–MS, P_AIS_: the peak area internal standard obtained, C_IS_: the concentration of internal standard and C_RS_: the concentration of reference standard.

### Quantification of fatty acid methyl esters

Quantification of fatty acids methyl ester was calculated from the peak area of a known amount of internal standard and a peak area of the analyte. All these analyses were performed in duplicate. The calculation is shown in Eq. 3 [32–34]:

3 $${\text{Concentration of FAME}} = {\text{C}}_{\text{FA}} \left( {{{\upmu{\text{g}}} \mathord{\left/ {\vphantom {{\upmu{\text{g}}} {\text{mL}}}} \right. \kern-0pt} {\text{mL}}}} \right) = \frac{{{\text{P}}_{\text{AFA}} \times {\text{C}}_{\text{IS}} }}{{{\text{P}}_{\text{AIS}} }} \times \frac{ 1}{\text{RRF}}$$

where P_AFA_: the peak area of the fatty acid, P_AIS_: the peak area of the internal standard, C_IS_: the concentration of the internal standard and C_FA_ is the concentration of coffee used for the analysis.

### Chemometric data analyses

Chemometric data analyses were performed using Minitab. Data were analyzed using correlation coefficient to test the effects of altitude on the fatty acid composition of the green coffee beans. One-way analysis of variance (ANOVA) was used to test for the presence of significant differences in the mean concentration of fatty acids among the topographical region (altitude) differences. The post hoc test (Tukey) was used to check if there were significant differences among the three regions. Differences were considered significant when p = 0.05. Box plot was used to show the distribution of fatty acids determined in the three topographic regions and chemical classes of volatile compounds of Ethiopian green coffee beans.

## Results and discussion

### Identification and quantification of fatty acids by GC–MS

**Fig. 1:**
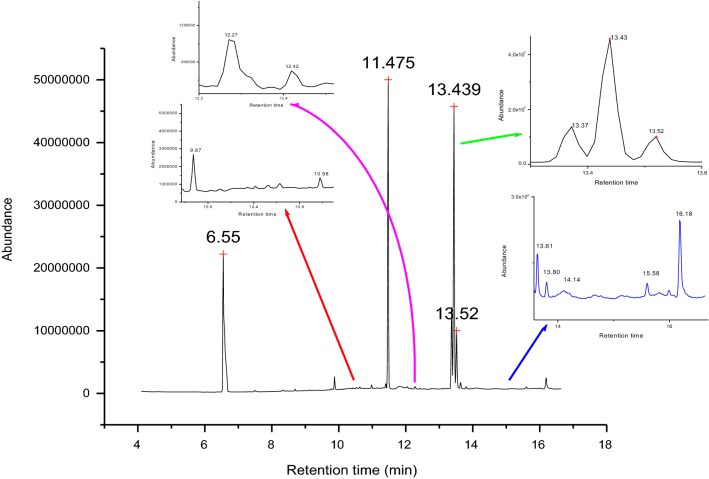
Chromatogram of the methyl esters of saturated and unsaturated fatty acids in green coffee beans detected by GC-MS

Figure 1 shows characteristics of fatty acids composition of Ethiopian green coffee beans analyzed by GC–MS. In the chromatogram, many saturated and unsaturated fatty acids were detected in all the green coffee beans. The fatty acid composition obtained in green coffee beans can be identified by comparing the retention times and mass spectral characteristics of the pure standard [14]. In this study, the detected composition of fatty acid was identified by matching with the mass spectrometric fragmentation pattern corresponding to the various peaks in the samples total ion chromatogram with the present mass spectral database in the instrument and relative retention time as listed in Table 1. In the past studies, many fatty acids were identified and quantified from Arabica coffee. They are palmitic, linoleic, stearic, oleic, arachidic, linolenic, palmitoleic and other trace fatty acids [14, 19, 20, 35, 36]. The above reported fatty acids were also detected in this study.

**Table 1:** The relative retention time (RRT) and method of identification of some fatty acids found in the Ethiopian green coffee beans

Number	Chemical name	Common name	RT	MS-ID	RRT
1	Tetradecanoic acid	Myristic acid	9.870	NIST-14	1.51
2	Pentadecanoic acid	Pentadecanoic acid	10.98	NIST-14	1.62
3	*cis*-7-Hexadecenoic acid	Hypogeic acid	11.16	NIST-14	1.70
4	Hexadecanoic acid	Palmitic acid	11.46	NIST-14	1.75
5	Heptadecanoic acid	Margaric acid	12.42	NIST-14	1.90
6	9-Octadecenoic acid	Oleic acid	13.35	NIST-14	2.04
7	9,12-Octadecadienoic acid	Linoleic acid	13.43	NIST-14	2.05
8	Octadecanoic acid	Stearic acid	13.61	NIST-14	2.06
9	9,12,15-Octadecatrienoic acid	Linolenic acid	13.78	NIST-14	2.08
10	Eicosanoic acid	Arachidic acid	16.18	NIST-14	2.47

RRT is a retention time relative to the decanoic acid

Fatty acids found in the studied 40 Ethiopian green coffee bean samples are given in Table 2. The major fatty acids of green coffee beans are linoleic (C18:2), palmitic (C16:0), stearic (C18:0), oleic (C18:1), arachidic (C20:0) and linolenic acids. In general, in green coffee beans, linoleic and palmitic acids are the dominant fatty acids. This is similar to the literature reported by Dong et al. [21]. The unsaturated fatty acid, linoleic acid, was the most abundant fatty acid and the second abundant fatty acid was palmitic acid [14, 21, 37]. However, in this study saturated fatty acid, palmitic acid with the average value of 55.5 mg/g (43.5%) was dominant and the second dominant was unsaturated fatty acid linoleic acid with the average value of 51.6 mg/g (39.4%). The other major fatty acids present in green coffee beans are oleic and stearic acids with the value of 8.92 mg/g and 12.3 mg/g, respectively. These are almost similar to the literature reported values [14, 36, 37]. Margaric, eicosanoic, myristic, pentadecanoic and other fatty acids were present in trace amount in green coffee beans of this work. The dominant fatty acids, linoleic and palmitic acids covered above 82% of the total fatty acid measured in the green coffee beans.

**Table 2:** The concentration of four main fatty acids (mg/g) in the green coffee beans; group 1 (1500–1700 m), group 2 (1701–1900 m) and group 3 (> 1900 m)

Sample region	Altitude (masl)	Palmitic acid (mg/g)	Stearic acid (mg/g)	Oleic acid (mg/g)	Linoleic acid (mg/g)
Group 1	1512	98.6 ± 2.7	8.53 ± 0.83	6.0 ± 0.45	38.3 ± 3.4
	1518	84.0 ± 2.1	21.3 ± 0.88	12.9 ± 0.90	93.7 ± 3.3
	1528	44.9 ± 4.8	23.6 ± 3.3	13.6 ± 0.75	82.9 ± 3.3
	1578	79.0 ± 5.1	18.5 ± 3.1	8.2 ± 0.14	59.0 ± 2.2
	1605	72.5 ± 3.5	16.7 ± 3.1	10.8 ± 1.3	64.5 ± 3.0
	1618	57.8 ± 1.8	16.2 ± 2.3	13.5 ± 1.68	96.2 ± 5.6
	1655	77.1 ± 5.4	9.93 ± 0.88	8.00 ± 0.29	50.9 ± 1.8
	1655	54.2 ± 2.7	10.1 ± 0.70	7.8 ± 0.79	45.1 ± 2.4
Group 2	1715	89.0 ± 3.9	10.4 ± 0.86	7.9 ± 0.57	47.3 ± 4.2
	1735	50.0 ± 3.4	14.5 ± 0.91	12.4 ± 1.1	71.1 ± 5.8
	1753	47.4 ± 3.5	26.1 ± 2.5	14.4 ± 1.2	66.3 ± 4.3
	1775	46.5 ± 1.9	12.3 ± 1.6	9.10 ± 0.27	67.8 ± 3.4
	1780	69.1 ± 5.3	6.86 ± 0.84	3.50 ± 0.17	36.3 ± 5.4
	1786	71.2 ± 3.2	10.60 ± 1.0	6.6 ± 1.4	40.5 ± 2.8
	1825	56.8 ± 4.4	8.02 ± 0.86	7.7 ± 0.92	33.6 ± 2.7
	1828	52.6 ± 1.8	9.20 ± 0.52	6.5 ± 0.90	47.6 ± 3.8
	1848	43.8 ± 1.8	12.7 ± 1.3	11.7 ± 0.98	70.2 ± 2.4
	1857	55.2 ± 3.0	9.37 ± 0.96	7.0 ± 0.14	42.8 ± 2.1
	1857	50.1 ± 3.2	16.2 ± 2.6	12.8 ± 1.2	73.5 ± 5.8
	1858	49.1 ± 3.8	8.66 ± 1.1	5.6 ± 0.68	36.9 ± 2.0
	1859	50.1 ± 3.3	23.0 ± 3.0	10.9 ± 0.71	36.0 ± 3.3
	1865	44.6 ± 2.2	14.3 ± 1.5	17.4 ± 0.16	85.1 ± 4.7
	1867	48.4 ± 2.7	9.56 ± 0.80	6.0 ± 0.18	42.5 ± 3.8
	1871	37.5 ± 2.6	3.03 ± 0.10	4.9 ± 0.0	40.4 ± 4.3
	1871	47.8 ± 3.0	7.24 ± 0.54	4.4 ± 0.21	35.1 ± 3.3
	1878	82.9 ± 4.1	9.60 ± 1.1	11.8 ± 0.37	46.4 ± 4.1
	1888	64.3 ± 4.6	19.1 ± 1.3	16.6 ± 0.77	86.2 ± 1.4
Group 3	1909	47.5 ± 3.8	23.5 ± 2.5	11.1 ± 0.81	25.2 ± 2.8
	1909	43.3 ± 4.5	9.66 ± 1.2	5.3 ± 0.05	49.2 ± 4.3
	1923	35.7 ± 2.9	11.5 ± 0.86	11.3 ± 0.69	50.6 ± 4.4
	1950	41.5 ± 5.7	13.5 ± 2.1	6.6 ± 0.7	42.4 ± 4.7
	1952	48.0 ± 3.8	8.04 ± 0.98	11.1 ± 1.1	25.6 ± 2.5
	1960	40.0 ± 1.6	8.91 ± 0.38	5.2 ± 0.15	39.6 ± 3.0
	1988	87.8 ± 3.7	8.55 ± 0.88	5.9 ± 0.68	42.8 ± 3.3
	1988	43.9 ± 2.7	3.63 ± 0.57	6.2 ± 0.40	42.4 ± 4.1
	1990	40.7 ± 3.6	7.71 ± 1.0	5.7 ± 0.14	39.9 ± 2.0
	1990	26.6 ± 1.9	8.00 ± 1.1	5.6 ± 0.18	29.3 ± 2.9
	1998	42.5 ± 4.3	8.53 ± 0.86	7.8 ± 0.90	38.3 ± 2.8
	1998	38.8 ± 2.5	7.47 ± 0.86	5.7 ± 0.58	40.4 ± 2.5
	2210	45.9 ± 4.0	11.1 ± 1.7	7.9 ± 0.69	45.8 ± 4.9
Average		55.5	12.3	8.92	51.6
Maximum		98.6	26.1	17.4	96.2
Minimum		26.8	3.03	3.54	25.2

Statistical analysis using one-way ANOVA (p = 0.05) was performed to test the presence of significant differences among the mean concentration of fatty acids in coffee beans from the three topographical regions (Table 3). Coffee beans from the group 1 contain a significantly higher amount of total fatty acid than the other two groups. In addition, results of one-way ANOVA (p = 0.05) indicated that the concentration of palmitic acid found in group 1 coffees is significantly different from that obtained from the other two groups (Table 3).

**Table 3:** Mean concentration (mg/g) of fatty acids found in the green coffee beans of the three topographical regions (minimum (min) and maximum (max) values are also included)

Topographical regions	Palmitic acid (mg/g)	Stearic acid (mg/g)	Oleic acid (mg/g)	Linoleic acid (mg/g)
Group 1 N = 8				
Mean	71.0^a^	15.6^a^	10.1^a^	66.3^a^
Min	44.9	8.53	6.00	38.3
Max	98.6	23.6	13.6	96.2
Group 2 N = 19				
Mean	55.6^b^	12.1^ab^	9.33^a^	52.9^ab^
Min	37.5	3.03	3.50	33.6
Max	89.0	26.1	17.4	86.2
Group 3 N = 13				
Mean	44.8^b^	10.0^b^	7.34^a^	39.3^b^
Min	26.6	3.63	5.20	25.2
Max	87.8	23.5	11.3	50.6

The same letters of superscript in the same column indicates non significance at p = 0.05 as determined by the Duncan’s multiple range test

Individually, the level of palmitic acid present in group 1 (mean 71.0 mg/g) green coffee beans is significantly higher than that present in the group 2 (mean 55.6 mg/g) and group 3 (44.8 mg/g) green coffee beans (p = 0.05). The other two fatty acids, linoleic and stearic acids are higher in group 1 and significant difference from the other two groups. But, there is no significant difference in linoleic and stearic acids between groups 2 and 3 (Table 3). While the values of oleic acid are not significantly different between groups 2 and 3 but its values are significantly different between groups 1 and 3 (Table 3).

Furthermore, the post hoc test (Tukey) was used to check if there is significant differences (at p = 0.05) in the major fatty acids contents of the green coffee beans among the three regions. The test showed that there is significant difference in palmitic acid between groups 1 and 2 and between groups 1 and 3. The test also showed that there is no significant differences (at p = 0.05) in the stearic and oleic acids in the three groups. While there is significant differences (at p = 0.05) in the linoleic acid between groups 1 and 3. However, there is no significant differences (at p = 0.05) in the linoleic acid between groups 1 and 2 and groups 2 and 3.

**Fig. 2:**
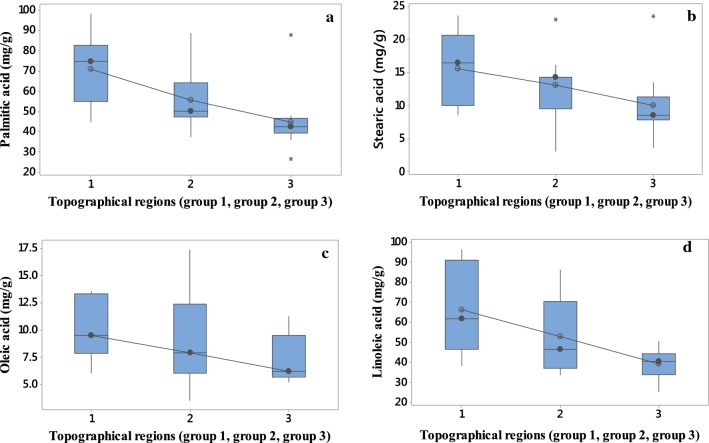
Boxplot showing the distribution of the concentration (mg/g) of palmitic acid (**a**), stearic acid (**b**), oleic acid (**c**) and linoleic acid (**d**) among green coffee beans from the three topographical regions. Horizontal bars indicate the median for each class; vertical bars indicate the maximum and minimum value. The stars present in **a** and **b** indicates possible outlier

Box plot distribution of fatty acid contents in 40 green coffee samples of the three topographical regions are shown in Fig. 2. Palmitic acid is an abundant content in green coffee beans and its distribution is a wider spread in the three topographical regions. However, oleic acid has a narrow spread in the three landscapes with lower mean value. Consequently, box plots distribution in Fig. 2 shows the range of fatty acids in green coffee bean samples of the three topographical regions.

The variations of fatty acid contents of green coffee beans can happen by genetic character, harvesting and postharvest processing methods, agricultural practices and growing environmental conditions [14]. In Ethiopia, agricultural practices are mainly of the traditional way of farming [29] and harvesting is normally performed by handpicking of ripe coffee cherries. Therefore, variances in agricultural practices and harvesting methods are doubtful to explain the variations in the fatty acid contents of the coffee samples. Regarding the postharvest processing conditions, the coffee samples were processed by dry processing methods. Among the limited literature in this regard, Joet et al. [36] have found that wet processing does not have an effect on the fatty acid composition of green coffee beans. On the other hand, several authors have indicated that the fatty acid composition of green coffee beans is mainly controlled by the mean air temperature during seed development [36], especially during the last 5 months before harvest [14]. Moreover, several investigators have indicated the significant influence of genetic properties on the fatty acid content of green Arabica coffee beans [14, 35]. Many authors have identified the existence of high genetic variability among coffees grown in different regions of Ethiopia. The variation in the fatty acid composition of the coffee samples is due to the variation in genetic properties, altitudes and environmental conditions of the coffee plants. Similarly, Villarreal et al. [14] have found significant variation in the fatty acid composition of green Arabica coffee beans from different regions of Colombia. The observed variation has also allowed for the topographical regions determination of the coffee beans.

The average concentration corresponding to each of the four main fatty acids determined in the three topographical regions samples was compared with the values reported for Arabica green coffee beans from different countries. In the literature, the concentration of fatty acid is expressed in terms of weight percent of the total fatty acids, and hence, in this study, the reported in mg/g is converted to percent by weight of the total fatty acids to simplify the comparison. Generally, the amounts of fatty acids found in Ethiopian green coffees beans are comparable with the green coffee beans of other countries listed in Table 4.

**Table 4:** Comparison of the concentration (%) of fatty acids found in green coffee beans with different countries

Fatty acids	Country of coffee origin					
	Colombia (n = 15)	Reunion Island (n not given)	Colombia (n = 42)	Unspecified	Unspecified (n = 40)	Ethiopia (n = 40)
Palmitic acid	32.0–35.0	35.0 ± 1.0	31.0–35.0	34.11–35.9	33.0–36.0	40.4–46.4
Linoleic acid	42.0–45.0	44.0 ± 2.0	41.0–46.0	42.10–44.5	43.0–46.0	37.0–42.0
Stearic acid	7.00–8.0	7.00 ± 1.0	6.00–8.00	5.98–6.80	7.00–9.00	8.20 –10.5
Oleic acid	9.00–10.0	7.00 ± 1.0	8.00–12.0	8.53–10.4	7.00–9.00	6.22–7.50
Reference	[29]	[30]	[14]	[21]	[20]	This study

The concentration of palmitic acid (40.4–46.4%) found in Ethiopian coffee beans are a higher than that of palmitic acid (31.0–35.0%) reported from the other countries. However, linoleic acid (37.0–42.0%) is lower than the linoleic acid (41.0–46.0%) reported from the other countries. In general, the average value of linoleic acid present in this study is lower than that reported value. The concentration of oleic and stearic acids found in Ethiopian green coffee beans is comparable with the reported values of 6.22–7.50% and 8.22–10.5% from Colombia and Reunion Island, respectively.

### Effect of altitude of the coffee plants on the four main fatty acids contents in green coffee beans

**Fig. 3:**
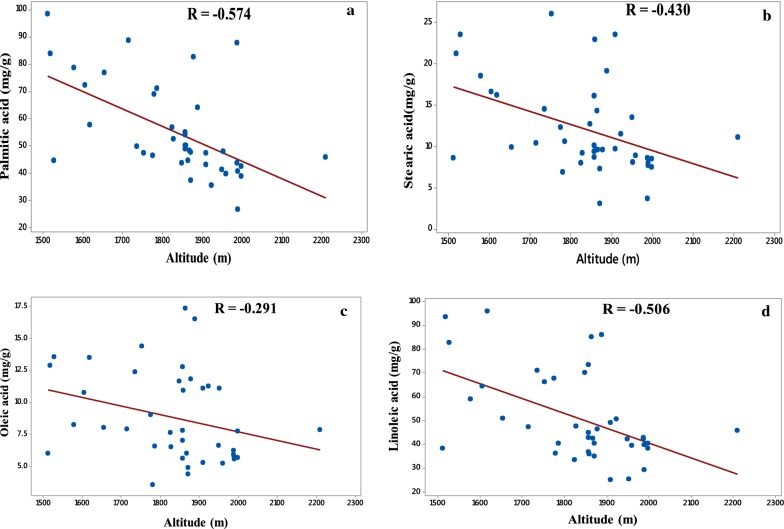
Scatter plot and correlation of the four main fatty acids in green coffee beans [palmitic acid (**a**), stearic acid (**b**), oleic acid (**c**) and linoleic acids (**d**)] versus the altitude of the coffee plants

The effect of altitude of the coffee plants on the fatty acid composition of the green coffee beans were studied by evaluating the correlation coefficients between four main fatty acids contents in green coffee beans and the altitude of the coffee plants. Figure 3 shows that palmitic (R = − 0.574), linoleic (R = − 0.506), stearic (R = − 0.43) and oleic acids (R = − 0.291) contents of green coffee beans are moderately negatively correlated with altitude of the coffee plants. The negative correlation indicates that the fatty acids contents decreases with increasing altitude of the coffee plants. The correlation coefficients of individual fatty acid are different from each other which clearly indicate that the altitude of coffee plants affects the composition of fatty acids in green coffee beans and hence affects the quality of coffee. The fatty acid composition of green coffee beans can also be used to determine the topographical origin of coffee plants. These observations are different from the only literature reported by Bertrand et al. [13]. They have observed that palmitic and linoleic acids are increased as elevation increased, but their study was up to the elevation of 1450 masl and they did not evaluate the correlation coefficients related with higher altitudes above 1450 masl. The chemical composition obtained in the green coffee bean is affected by soil type, climatic condition, variety type, elevation (altitude) and location [25]. A total lipid content of the green coffee beans was also significantly correlated with altitude [35]. In addition, caffeine and chlorogenic acid concentrations were increased with increasing elevation above 1200 masl and fat contents were increased with increasing elevation, but it decreased at the highest elevations. In general, elevation did not affect the fat concentration [13].

The correlation coefficients of four fatty acids with each other in 40 green coffee bean samples are listed in Table 5. When correlated with each other, strong correlation was observed, particularly between stearic and oleic acids (R = 0.727), stearic and linoleic acids (R = 0.555), and oleic and linoleic acids (R = 0.742) have a positive significant correlation at (*p* = 0.01). Nevertheless, there were very weak positive correlation between linoleic and palmitic acids, palmitic and stearic acids and palmitic and oleic acids.

**Table 5:** The correlation value of four fatty acids in green coffee beans with each other at significant level (*p* = 0.01)

Fatty acids	Palmitic acid (mg/g)	Stearic acid (mg/g)	Oleic acid (mg/g)	Linoleic acid (mg/g)
Palmitic acid (mg/g)	1.00	0.09	0.04	0.139
Stearic acid (mg/g)	0.09	1.00	0.727**	0.555**
Oleic acid (mg/g)	0.04	0.727**	1.00	0.742**
Linoleic acid (mg/g)	0.139	0.555**	0.742**	1.00

** Correlation is significant at the 0.01 level (1-tailed)

## Conclusion

The major fatty acids (palmitic, stearic, linoleic and oleic acids) of the green coffee beans showed moderate negative correlation with the altitude of coffee plants grown in Ethiopia. The negative correlation indicates that the fatty acids contents decreases with increasing altitude of the coffee plants. Accordingly elevation of coffee plants affects the fatty acids (palmitic, stearic, linoleic and oleic acids) contents of green coffee beans and hence affects the quality of coffee. The fatty acid composition of green coffee beans can also be used to determine the topographical origin of coffee plants. A significant difference in the content of palmitic acid in the green coffee beans was observed in the topographic regions of the coffee plants. In addition, linoleic, stearic and oleic acids have a moderate to strong correlation with each other. However, palmitic acid has a weak positive correlation with the three fatty acids. In green coffee beans, palmitic and linoleic acids are the major fatty acids with the average value of 55.5 mg/g and 51.6 mg/g, respectively. In general, the fatty acids contents in the Ethiopian green coffee beans are comparable to that in the other countries.

## Data Availability

The data sets used and analysed during the study are available to readers as in the manuscript. There are no additional data with the authors. All the data are included in the manuscript.

## Abbreviations

m.a.s.l.: Meters above sea level
GC–MS: Gas chromatography–mass spectrometry
R: Correlation coefficient
^1^H NMR: Proton nuclear magnetic resonance
^13^C NMR: Carbon thirteen nuclear magnetic resonance
SNNPR: South Nations Nationalities and Peoples Region
FAMEs: Methyl esters of fatty acids
RRF: Relative response factor
P_ARS_: Peak area of the reference standard
P_AIS_: Peak area internal standard
C_IS_: Concentration of internal standard
C_RS_: Concentration of reference standard
ANOVA: Analysis of variance
RT: Retention time
RRT: Relative retention time
MS-ID: Mass spectrometry identification
